# Glucocorticoids Regulate Circadian Rhythm of Innate and Adaptive Immunity

**DOI:** 10.3389/fimmu.2020.02143

**Published:** 2020-09-18

**Authors:** Akihiro Shimba, Koichi Ikuta

**Affiliations:** ^1^Laboratory of Immune Regulation, Department of Virus Research, Institute for Frontier Life and Medical Sciences, Kyoto University, Kyoto, Japan; ^2^Human Health Sciences, Graduate School of Medicine, Kyoto University, Kyoto, Japan

**Keywords:** circadian rhythm, glucocorticoid, IL-7 receptor, inflammatory cytokine, T cell

## Abstract

Animals have evolved circadian rhythms to adapt to the 24-h day-night cycle. Circadian rhythms are controlled by molecular clocks in the brain and periphery, which is driven by clock genes. The circadian rhythm is propagated from the brain to the periphery by nerves and hormones. Glucocorticoids (GCs) are a class of steroid hormones produced by the adrenal cortex under the control of the circadian rhythm and the stress. GCs have both positive and negative effects on the immune system. Indeed, they are well known for their strong anti-inflammatory and immunosuppressive effects. Endogenous GCs inhibit the expression of inflammatory cytokines and chemokines at the active phase of mice, regulating the circadian rhythm of tissue inflammation. In addition, GCs induce the rhythmic expression of IL-7R and CXCR4 on T cells, which supports T cell maintenance and homing to lymphoid tissues. Clock genes and adrenergic neural activity control the T cell migration and immune response. Taken together, circadian factors shape the diurnal oscillation of innate and adaptive immunity. Among them, GCs participate in the circadian rhythm of innate and adaptive immunity by positive and negative effects.

## Introduction

Circadian rhythms are endogenously regulated by molecular clocks, which can be controlled by the 24 h day-night cycle. Light and dark stimuli are converted into neuronal activity in the retina and transmitted to the suprachiasmatic nucleus (SCN) of the brain. This neuronal activity induces neuropeptide production in the brain, which stimulates the synthesis of hormones in endocrine organs. Together with sympathetic neurons, the hormones integrate circadian rhythms in peripheral organs. The circadian rhythm controls physiological activities in the body, such as metabolism in liver, blood pressure, sleep, and immune response. Especially, immune functions such as cytokine expression, leukocyte mobilization, and antigen priming show diurnal changes. Past studies have revealed that clock genes, nervous signals, and hormones control the circadian rhythm of the immune system. Especially, glucocorticoids (GCs), a group of steroid hormones produced by the adrenal cortex, affect the circadian rhythm of the immune system. In this article, we will first explain the general functions of GCs in the circadian control of the immune system, then introduce our recent works, and finally discuss potential interactions between GCs and other circadian mediators in immune regulation.

## Glucocorticoids and Circadian Rhythms

At steady state, GC production is under the control of circadian rhythms (1). Blood GC levels oscillate with a peak in early morning and nadir at night in diurnal animals like humans and in reverse fashion in nocturnal animals like rodents. Neuronal activity in the SCN induces the paraventricular nucleus (PVN) of the hypothalamus to secrete corticotropin-releasing hormone (CRH). CRH then stimulates the anterior pituitary, leading to the production of adrenocorticotropic hormone (ACTH) in the blood. ACTH finally stimulates the adrenal cortex to produce GCs. In addition, GCs are also induced by endogenous and physiological stress (1).

Glucocorticoids exert their functions through binding to intracellular GC receptor (GR) (2). GR consists of an N-terminal activation function-1 (AF-1) domain, DNA-binding domain (DBD), and ligand-binding domain. GC-GR binding induces GR to dimerize, translocate to the nucleus, and bind to specific DNA sequences, known as GC-response elements (GREs), to activate or repress the transcription of target genes. Based on the gene response, GREs are classified as positive or negative (nGREs). Because mineralocorticoid receptor (MR) and GR are related steroid hormone receptor and share their basic structure of nuclear receptor, GCs can bind to both of GR and MR, suggesting that endogenous GCs work through GR and MR to regulate immunity (3). In addition, GR directly interacts with other transcription factors, such as NF-κB, AP-1, MAPK, and STAT, to regulate their functions.

The molecular clock controls circadian rhythm by transcription-translation feedback loop of clock genes (4, 5). The core clock components, BMAL1 and CLOCK, form a heterodimeric complex that stimulates the transcription of E-box containing genes of other molecular clock components, including PERs, CRYs, REV-ERBs, and RORs. As the core clock loop, the negative feedback in which PER/CRY complex repress their own transcription by interacting with BMAL1/CLOCK complex drives the 24-h oscillation of PER/CRY expression. Additionally, REV-ERBs and RORs are parts of the accessory loop that represses or induces the expression of BMAL1 and NFIL3. NFIL3, as the downstream mediator of the molecular clock, induces the expression of PERs, REV-ERBs, and RORs. GCs also regulate the expression of some clock components such as PER1, PER2, REV-ERBα, and NFIL3 (6–8). Especially, GRE motifs are present in *Per1, Per2*, and *Nfil3* gene loci (6). So, central nervous system directs the diurnal production of GCs, and GCs have function to integrate the circadian rhythm of peripheral organs.

The diurnal cycle of GCs also modulates metabolism. Because GR directly induces the expression of metabolic enzymes such as phosphoenolpyruvate carboxylase 1 (PCK1) and glucose-6-phosphatase catalytic subunit (G6PC), GCs enhance gluconeogenesis in the liver and lipolysis in adipose tissues to release fatty acid and glycerol (9). Moreover, GCs can enhance the effects of adrenergic neural activity, known as permissive effects. For example, GCs increase the sensitivity of skeletal muscle to adrenaline (10). GCs also enhance glucagon-, adrenaline-, and cyclic AMP-mediated gluconeogenesis; these metabolic pathways are attenuated in adrenalectomized mice (9). Therefore, the circadian oscillation of GCs induces a diurnal change of metabolic activity in peripheral organs.

## Immunosuppressive Effects

Glucocorticoids help regulate the circadian expression of a number of inflammatory cytokines. GCs are well known for their strong anti-inflammatory and immunosuppressive effects and are thereby widely used for treatment of inflammatory and autoimmune diseases (11, 12). First, GR carries out immunosuppressive roles by activating or suppressing gene expressions via binding to GREs. For example, GR transactivates anti-inflammatory factors, GILZ and IκBα, whereas GR suppresses the expressions of inflammatory cytokines and factors such as IL-6 and C3 through nGRE (13). Second, GR can suppress the inflammatory factor expression by interacting directly with other transcriptional factors such as NF-κB and AP-1 as transrepression. GR interacts with DNA-bound NF-κB and AP-1 and suppresses their activities by recruiting the transcription repressor such as nuclear receptor corepressor and by inhibiting the recruitment of transcriptional coactivators such as nuclear coactivator and the detachment from DNA (14, 15). In addition, recent studies reported other mechanisms that GR regulates the transcription of AP-1 via GR-binding to half-site of cryptic GRE embedded in AP-1 response elements (TREs) (16), and DBD of GR can directly bind in a DNA-dependent manner to NF-κB response elements (17). By these mechanisms, GCs inhibit the synthesis of various cytokines including IL-1, IL-2, IL-4, IL-5, IL-6, IL-12, IL-18, GM-CSF, TNF-α, and IFN-γ (18–21).

Researchers have also showed *in vivo* effects of GCs to suppress immune responses in rodents. In a mouse model of inflammation, LPS-stimulated GR-deficient macrophages show a higher expression of several inflammation-associated genes such as IL-6, TNF-α, and COX-2, leading to lethality (22). This effect is because GR directly associates with p38 MAPK and inhibits the latter’s activity through MAPK phosphatase-1 induction. Like macrophages, GCs also suppress the responses of dendritic cells (DCs). DC-specific (CD11c-Cre) GR-deficient mice produce higher amounts of the inflammatory cytokines IL-1β, TNF-α, and IL-12 (23). In lymphocytes, GCs suppress Th1 cell differentiation and reduce IFN-γ production by Th1, CD8 T, and NK cells, leading to the inhibition of cytotoxic responses (24–26). The deficiency of GR causes the increased production of IFN-γ from Th1 and NK cells to lead the higher mortality in the infection of toxoplasma and mouse cytomegalovirus (27–29).

Glucocorticoids are partly involved in regulation of the circadian rhythm of immune responses by clock genes. The expression of inflammatory cytokines by innate immune cells after LPS stimulation exhibits circadian oscillation due to diurnal change of the expression of TLR and clock genes (5). Wang et al., reported that circadian oscillation of response to endotoxin was disrupted in *Per2* mutant (Per2m) mice and that the Per2m mice showed alleviated endotoxin shock (30, 31). Interestingly, corticosterone concentration became higher in Per2m mice after LPS administration, and the increased corticosterone might have suppressed the inflammation and mortality of Per2m mice. They showed that the upregulation of CLOCK and BMAL1 due to *Per2* mutation induced the expression of steroidogenic acute regulatory protein (StAR), a rate-limiting enzyme in GC synthesis, by binding to the promoter of *Star* gene. Thus, the molecular clock can control the circadian rhythm of inflammation by regulating GCs synthesis.

Glucocorticoids also control the circadian rhythm of chemokine expression. Gibbs et al., found that CXCL5, a chemokine recruiting neutrophils and monocytes at the inflammatory site, is produced in mice in a diurnal fashion under the control of diurnal GC secretion (32). CXCL5 expression is suppressed through GR binding to nGREs in the CXCL5 promoter. In mice with lung inflammation induced by LPS, CXCL5 expression shows a diurnal oscillation with a peak at daytime, triggering the diurnal accumulation of neutrophils in the lung. The levels of inflammatory cytokines such as IL-6, TNF-α, and G-CSF increase shortly after the peak of CXCL5 and neutrophil count. Because GCs shows an anti-phase diurnal oscillation with CXCL5 and neutrophils, neutrophil migration might be reduced at night via the repression of CXCL5 expression by GCs. In fact, the Gibbs study further reported that the circadian change of CXCL5 and neutrophils is impaired in adrenalectomized mice. BMAL1 is also involved with circadian rhythm of inflammation via macrophages and neutrophils (33). Although a circadian change of CXCL5 was not affected in airway club cell-specific (CCSP-Cre) BMAL1-deficient mice, the diurnal change of neutrophil infiltration into lung was impaired, and inflammation was enhanced, suggesting that BMAL1 in club cells plays some role in controlling circadian rhythm of neutrophil migration and immunosuppression independently of CXCL5 (32). The study also found that the daytime increase of neutrophils efficiently protects mice against bacterial infection by *S. pneumoniae.* However, the same group reported later that the airway club cell-specific deletion of GR caused a normal circadian rhythm of LPS-mediated neutrophil infiltration despite the loss of CXCL5 circadian rhythm (34). Additionally, macrophage-specific (LysM-Cre) deletion of GR resulted in the loss of the circadian oscillation of CXCL5 but not of neutrophils. These reports indicate that GCs regulate the circadian rhythm of CXCL5 expression in lung club cells and myeloid cells, but that neutrophil infiltration is not regulated by GCs. Nevertheless, there are mechanisms of neutrophil migration in the lungs that still need to be worked out.

## Immune-Enhancing Effects

In addition to their negative effects, GCs can have positive effects on the immune system. Wiegers et al., reported that cytokine receptors for IL-2, IL-6, IFN-γ, GM-CSF, and TNF-α were potently upregulated in various types of cells by GCs (35), and Franchimont et al., reported that dexamethasone (DEX) treatment enhanced the expression of IL-7 receptor α-chain (IL-7Rα) in human blood T cells (36). IL-7 is a cytokine essential for the development and maintenance of lymphocytes (37–39). IL-7R consists of the IL-7Rα chain and common γ-chain, and induces the phosphorylation of STAT5 and PI3 kinase. This signal transduction promotes the survival and proliferation of T cells, B cells, and innate lymphoid cells (ILCs), and supports their maintenance in peripheral lymphoid organs. Furthermore, IL-7 rescues T cells from apoptosis induced by GCs, and IL-7Rα upregulated by DEX enhances IL-2Rα expression and IL-2–induced survival (36).

To investigate the mechanism how GCs regulate IL-7Rα expression, Lee et al., searched the IL-7Rα locus and found DNA sequences conserved among different species at 3.6 kb upstream of the promoter (40). They designated this region non-coding conserved sequence 1 (CNS-1). CNS-1 is about 300 bp long and has 86% homology between human and mouse. It also has two GRE motifs and one NF-κB motif that are conserved. Point mutations in GREs impair transactivation of the IL-7Rα promoter by GCs. Later, Abe et al., generated IL-7Rα CNS-1 deletion mice and tested the *in vivo* role of CNS-1 in IL-7Rα expression (41). Although IL-7Rα expression was unchanged in thymocytes, CNS-1-deletion mice exhibited a reduction of IL-7Rα expression in peripheral T cells, the absolute numbers of which were decreased in spleen and lymph nodes. Moreover, IL-7Rα induction by GCs was lost in T cells of CNS-1 deletion mice. Thus, CNS-1 is a proximal enhancer of the IL-7Rα locus regulated by GCs.

**FIGURE 1:**
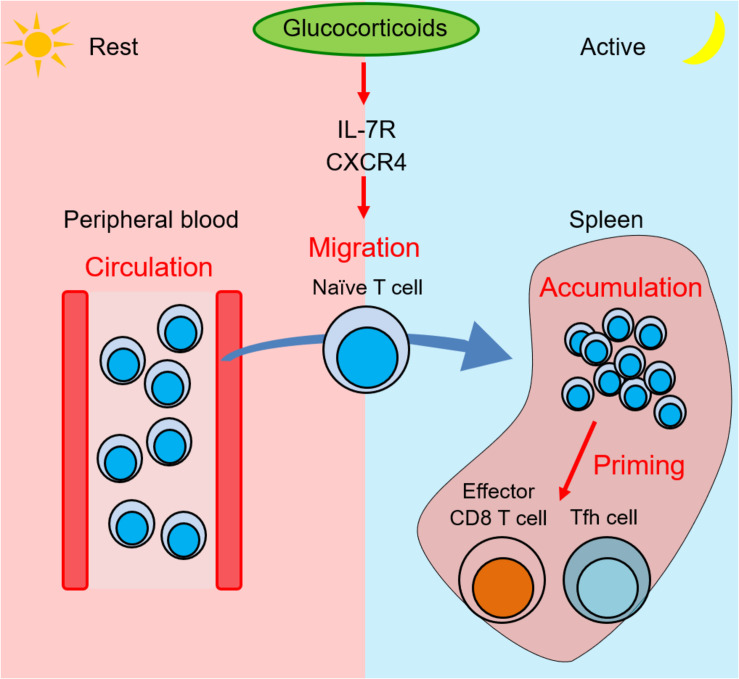
Glucocorticoids induce T cell migration into spleen from blood and enhance immune response at night by expression of IL-7R and CXCR4. Zeitgeber time (ZT) is a unit of cycle consisted of 12 h light/12 h dark phase. ZT0 is defined as the time when light on and ZT12 is defined as the time when light off. At the rest phase (ZT4) in mice, more T cells circulate in peripheral blood. At the onset of the active phase, the adrenal cortex releases glucocorticoids, which induce IL-7R and CXCR4 expression on T cells. At the active phase (ZT16), the elevated receptors trigger T cell migration into second lymphoid tissues such as spleen, lymph nodes, and Peyer’s patches. The T cell accumulation induces a stronger immune response by effector CD8 T cells and follicular helper T (Tfh) cells against bacterial infections and soluble antigens.

To clarify the biological significance of IL-7Rα induction by GCs, we generated the mice harboring point mutations in two GREs of the CNS-1 (GRE point mutant mice, GREm mice) and analyzed them together with T cell-specific (CD4-Cre) GR-deficient mice (42). IL-7Rα expression on T cells is elevated at night and diminished at daytime in control mice, which is consistent with blood GC levels. Human blood T cells also show a diurnal change (43). Of interest, mouse T cells accumulate in secondary lymphoid organs, such as spleen, lymph nodes, and Peyer’s patches at night. By contrast, blood T cells accumulate at day. These results suggest that T cell recirculation between secondary lymphoid organs and blood has a diurnal oscillation. This diurnal oscillation is lost in T cell-specific GR-deficient and GREm mice. GC rhythms have also been linked to the diurnal change of T cell blood counts in humans. It was shown that cortisol induces chemokine receptor CXCR4 and that CXCR4 expression was reduced by mifepristone, an antagonist of steroid hormone receptors including GR, or by metyrapone, an inhibitor of GC synthesis, impairing the diurnal oscillation of T cells in human blood (44, 45). The observation supports the idea that the involvement of CXCR4 as a mediator for the effects of GCs on T cells is conserved both in humans and rodents. In addition, Besedovsky et al., showed that administration of spironolactone, an antagonist for mineralocorticoid receptor (MR), increased T cell counts in human peripheral blood at night. Because MR has the potential to bind to GCs, it suggests that GCs at low concentrations bind to MR and control the circadian rhythm of T cell distribution (46). CXCR4 expression on T cells shows a diurnal oscillation similar to IL-7Rα in control mice, but this pattern is abolished in T cell-specific GR-deficient and GREm mice. Additionally, the diurnal change of the T cell distribution is lost in T cell-specific CXCR4-deficient mice, indicating that IL-7Rα induced by GCs is responsible for the diurnal rhythm of the T cell distribution. IL-7 also has the potential to induce CXCR4 expression on T cells (47). These results suggest that the diurnal change of GCs triggers the diurnal distribution of T cells (Figure 1).

The change of T cell accumulation in lymphoid organs during course of the day might affect immune responses against foreign antigens. Such a diurnal change of immune responses is observed in innate and adaptive immunity. Our data showed that T cell accumulation in lymphoid organs induced by GCs at night enhances the efficiency of effector CD8 T cells against bacterial infection, and follicular helper T (Tfh) cells, germinal center B cells, and class-switched B cells against soluble antigens (Figure 1). Similarly, we also reported that T cell accumulation at night is observed in Peyer’s patches, where GR enhances germinal center formation and immunoglobulin class switching. Because it was reported that immune responses against *Salmonella* infection in gut is strongly induced at night (48), our findings imply that T cell accumulation in Peyer’s patches at night might contribute to bacteria removal in mice.

GC receptor also influences the differentiation and function of helper T cell subsets. Correlated with these points, Th1 cell differentiation *in vitro* is increased in T cells of T cell-specific GR-deficient mice. By contrast, it is reported that GR promotes Th2 cell differentiation (49). Furthermore, GCs have the potential to promote the production of Th2 cytokines. In culture, primed CD4 T cells pretreated with DEX produce higher levels of IL-4, IL-10, and IL-13 (50). We also reported that the Th2 differentiation of GR-deficient T cells is impaired in Th2-skewed culture. *Nfil3* is a clock gene that promotes the production of Th2 cytokines (51). The *Nfil3* gene locus contains a GRE motif (6), and its expression is reduced in GR-deficient T cells in the early phase of Th2 differentiation. Moreover, the expression of IL-4 in Tfh cells by immunization with a soluble antigen is reduced in T cell-specific GR-deficient mice (42). These mice show reduced levels of plasma IgG1 and IgG2b, which are regulated by IL-4. After priming, Th2 cells become memory Th2 cells and stay long in the periphery (52). Memory Th2 cells cause chronic allergies, such as atopy and asthma (53). GCs also drive the circadian rhythm of IL-7Rα expression in memory Th2 cells (42). On the other hand, the diurnal rhythm of IL-7Rα and the maintenance of memory Th2 cells are impaired in T cell-specific GR-deficient and GRE mutant mice (42). Because GCs induce IL-7Rα expression in memory Th2 cells and IL-7 is important for the homeostasis of memory T cells, IL-7R induction by GCs likely supports the survival of memory Th2 cells. Taken together, GCs promote the differentiation of naive T cells into Th2 cells and the maintenance of memory Th2 cells.

Past studies have revealed that pathogenic memory Th2 cells play an important role in allergic response in asthma (53). Allergic responses in lung and skin exhibit time-dependent manner of symptoms. Nakamura et al., showed that mast cell-mediated diurnal rhythm of cutaneous anaphylactic reaction is dependent on clock genes and adrenal gland, and such rhythm is ablated in Clock mutant, *Per2* mutant, and adrenalectomized mice (54–57). Interestingly, they found that GCs not only suppressed the inflammation but also modulated the rhythm of clock genes expression, affecting the diurnal cycle and responsiveness (56, 57). On the other hand, mutation of clock genes impaired the diurnal cycle of GC production and GC-mediated suppression of mast cell activation (54, 55). Thus, clock genes and GCs mutually influence each other, and control the diurnal allergic reaction. However, there are few studies on the relationship between clock genes and the function of Th2 cells. Because Th2 cells might have resistance to the suppressive effects of GCs, the role of GCs on Th2 cell function remain unclear. So, it is to be clarified whether diurnal oscillation of GCs and clock genes shapes the circadian rhythm of allergic response by promoting the maintenance and function of pathogenic Th2 cells.

## Relationship Between GCs and Other Circadian Factors on Immune Regulation

Because GCs enhance the homing and response of T cells, GCs might suppress innate immunity but enhance adaptive immunity. Similar to GCs, adrenergic receptors and the clock component BMAL1 in T cells control diurnal changes of the T cell distribution in lymph nodes, lymph, and blood. Druzd et al., found that the CCR7 expression on T and B cells show circadian oscillation with the peak at the active phase, but that the circadian oscillation of S1PR1 expression shows its peak at the rest phase in mice (58). BMAL1 enhances the CCR7-mediated recruitment of T and B cells to the lymph nodes and S1PR1-mediated egress. This mechanism leads to the circadian oscillation of the autoimmune response in an EAE model, in which Th17 cell number and symptoms were elevated at the active phase. Suzuki et al., showed that the signal from the adrenergic receptor, β2AR, augments the responsiveness of chemokine receptors CXCR4 and CCR7, and retains T and B cells in lymph nodes (59). The β2AR signal also enhances the differentiation of Tfh cells and immunoglobulin production by activated B cells. These effects lead to diurnal changes of the lymphocyte distribution and immune response. Thus, these studies suggest that adaptive immune responses are under the control of circadian rhythms through GCs, adrenergic receptor, and BMAL1.

In addition to the T cell distribution, other mechanisms also control the circadian rhythm of immune response. First, antigen presentation is important for the circadian rhythm of T cell response. Silver et al., reported that the expression of TLR9, CD80, and CD86 is high at the active phase, and CpG-OVA stimulation at the active phase efficiently induced the proliferation and cytokine production of T cells (60). Additionally, Nguyen et al., described circadian rhythm of monocyte distribution that the count of monocytes in spleen fluctuated during the course of the day with a peak at zeitgeber time (ZT)8 (61). Immune response against *L. monocytogenes* was induced strongly at ZT8 compared to ZT0, and BMAL1 regulated the circadian changes of the distribution, cytokine production, and bacterial rejection by monocytes. Sengupta et al., showed that BMAL1 regulated the diurnal difference of inflammation against influenza A virus which is induced strongly at the active phase via the monocyte infiltration in lung (62). Next, Fortier et al., found that T cell proliferation after anti-CD3 antibody stimulation became higher at the late rest or the active phase (circadian time (CT) 10-14) in a circadian manner, probably because of oscillating ZAP-70 expression with peak at CT 8 (63). It implies that intrinsic responsiveness of T cells follows the circadian rhythm. Same group also showed the antigen-presentation by OVA-pulsed DCs *in vivo* is more efficient at the rest phase (CT6) compared to at the active phase (CT18) (64). The circadian rhythm of T cell response persists even when using Bmal1-deficient DCs, whereas T cell-specific BMAL1-deficient mice lose the rhythm completely. Therefore, DC rhythms may contribute to some extent to the T cell response rhythm, but T cell-intrinsic rhythms are the main mechanism involved. Taken together, the circadian rhythm of T cell reactivity seems to show different time courses depending on situations. Thus, more studies will be needed to clarify the mechanisms of the circadian rhythm of immunity.

## Conclusion

**FIGURE 2:**
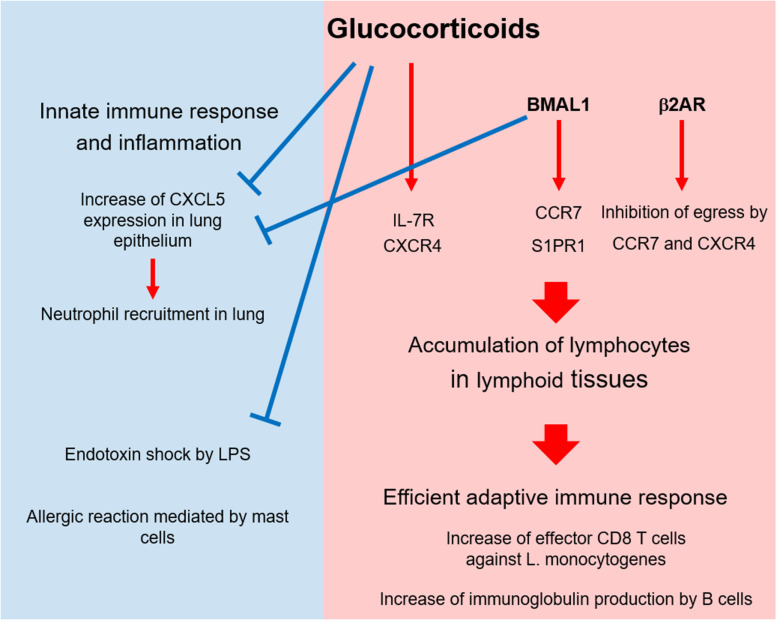
Circadian control of immunity by glucocorticoids and other factors. At the active phase, glucocorticoids (GCs), and BMAL1 suppress CXCL5 expression in lung epithelium and suppress neutrophil recruitment. In addition, GCs alleviate the endotoxin shock stimulated by LPS and the allergic response mediated by mast cells in skin and lung. As immune-enhancing effects, GCs, BMAL1, and β2 adrenergic receptor (β2AR) trigger T cell migration to and retention in lymphoid tissues by enhancing the expression and function of chemokine receptors at the active phase. This accumulation augments T cell-mediated immunity such as anti-bacterial response, B cell activation, and autoimmunity.

Generally, GCs suppress inflammation and trigger the descending phase of the circadian oscillation of innate immune responses. By contrast, GCs drive the circadian change of T cell distribution and response by inducing the expression of cytokine and chemokine receptors (Figure 2). Because other circadian factors such as clock genes and adrenergic signals promote the circadian cycles of an immune response, GCs might cooperate with them to enhance the immune response at the active phase. In addition, because GCs have the potential to enhance permissive effects, they might play a major role in controlling the circadian rhythm of immunity. Because infection risk increases during the active phase, it is reasonable that GCs and other factors induce T cells to home to lymphoid tissues and prepare for an immediate response against infection. Thus, when the circadian rhythm of GCs is disrupted, immune function might be impaired. Conversely, because GCs have the potential to enhance Th2 cell-mediated responses and allergies, their abnormal production might cause excessive Th2 response and allergy. In addition, GCs might affect the diurnal rhythm of asthma symptoms via mast cells and Th2 cells. Future work should focus on revealing the mechanism through which the irregular production of GCs causes immune dysfunction and inflammation such as allergy and autoimmunity.

## Author Contributions

AS wrote the first draft of the manuscript. KI modified, revised, and approved the submitted version. Both authors contributed to the article and approved the submitted version.

## Conflict of Interest

The authors declare that the research was conducted in the absence of any commercial or financial relationships that could be construed as a potential conflict of interest.
